# Analyze COVID-19 CT images based on evolutionary algorithm with dynamic searching space

**DOI:** 10.1007/s40747-021-00513-8

**Published:** 2021-09-06

**Authors:** Yunhong Gong, Yanan Sun, Dezhong Peng, Peng Chen, Zhongtai Yan, Ke Yang

**Affiliations:** 1 College of Computer Science, Sichuan University, Chengdu, 610065 China; 2 Shenzhen Peng Cheng Laboratory, Shenzhen, 518052 China; 3 Chengdu Ruibei Yingte Information Technology Co., Ltd, Chengdu, 610054 China; 4 Sichuan Zhiqian Technology Co., Ltd, Chengdu, 610041 China; 5 School of Computer and Software Engineering, Xihua University, Chengdu, 610039 China; 6 China National Tobacco Corporation Sichuan Province Co., Chengdu, 610000 China

**Keywords:** Evolutionary algorithms, Variable-length chromosomes, COVID-19, Batch normalization

## Abstract

The COVID-19 pandemic has caused a global alarm. With the advances in artificial intelligence, the COVID-19 testing capabilities have been greatly expanded, and hospital resources are significantly alleviated. Over the past years, computer vision researches have focused on convolutional neural networks (CNNs), which can significantly improve image analysis ability. However, CNN architectures are usually manually designed with rich expertise that is scarce in practice. Evolutionary algorithms (EAs) can automatically search for the proper CNN architectures and voluntarily optimize the related hyperparameters. The networks searched by EAs can be used to effectively process COVID-19 computed tomography images without expert knowledge and manual setup. In this paper, we propose a novel EA-based algorithm with a dynamic searching space to design the optimal CNN architectures for diagnosing COVID-19 before the pathogenic test. The experiments are performed on the COVID-CT data set against a series of state-of-the-art CNN models. The experiments demonstrate that the architecture searched by the proposed EA-based algorithm achieves the best performance yet without any preprocessing operations. Furthermore, we found through experimentation that the intensive use of batch normalization may deteriorate the performance. This contrasts with the common sense approach of manually designing CNN architectures and will help the related experts in handcrafting CNN models to achieve the best performance without any preprocessing operations

## Introduction

At the beginning of 2020, the outbreak of an atypical and person-to-person transmissible pneumonia caused by severe acute respiratory syndrome coronavirus 2 (SARS-COV-2, also known as COVID-19) spread worldwide. After a year, COVID-19 is still spreading, and there were more than 55.56 million confirmed cases and 1.33 million deaths worldwide as of November 10, 2020 [1]. As a practical and sensitive modality, radiological computed tomography (CT) is widely used to diagnose COVID-19 pneumonia, even in asymptomatic individuals. It is considered a particularly important screening tool to help doctors diagnose patients who have false-negative reverse transcription-polymerase chain reaction results. However, it is difficult for radiologists to distinguish COVID-19 cases from other similar viral pneumonia CT images, such as SARS. This is because they often overlap with other infectious and inflammatory lung diseases.

At present, data engineering experts are devoting to utilizing artificial intelligence technology to handle the difficult COVID-19 diagnosis task and save more lives [2, 3]. With the development of deep learning, thousands of neural network algorithms have been proposed in recent decades. Because of hierarchically abstract representations with local operations, convolutional neural networks (CNNs), as one of the main categories, are widely adopted in computer vision applications. Compared with standard neural networks, the critical advantage of the feature sharing mechanism in CNNs allows them to rely on far fewer weights and variables. By reducing the number of weights and variables that need to be learned, the complexity of the network is decreased, and the image generalization ability is improved. Moreover, pooling operations allow CNN architectures to be less affected by small variations in position. CNNs have been successfully applied to object detection [4], biological image classification [5], cross-modal retrieval [6] and other visual recognition tasks. Since LeNet-5 [68] was proposed in 1989, a series of CNNs variants have been developed, such as AlexNet [7] which uses dropout [8] to avoid overfitting, VGGNet [9], which trains a deeper neural network by $3\times 3$ filters throughout the network for considerable scale image recognition, and ResNet [10] whose main contribution lies in its reliance on residual learning by skip connections. Due to the enormous efforts of medical experts and the careful designs of statistical researchers, significant progress has been made to automatically interpret CT images and predict whether or not the CTs are positive for COVID-19 through deep learning [11], and the burden of medical professionals in reading CT scans has been alleviated.

While deep learning techniques are well developed, applying them to COVID-19 presents several challenges. First, the lack of clear rules regarding the hyperparameters, including the filter sizes and the choice of pooling function, consumes considerable time in designing architectures and hampers its widespread application. As a result, neural network architectures cannot quickly be properly designed according to the actual situation. Another inevitable limitation is that only a few data sets are publicly available, and almost all the data come from a few sources, mainly in China. As data might not represent the global population, model bias will be generated, which will lead to poor performance [12–14]. Finally, most of existing CNNs are problem-specified, and model architectures need to be redesigned according to different characteristics. Currently, many researchers are attempting to automate yield hyperparameter settings, which are competitive with those manually designed by deep learning experts. Neural architecture search (NAS) is a technology that designs the proper CNN architectures without intensive human effort. Numerous NAS strategies are used to explore the space of hyperparameter settings, including random search [15–17], Bayesian optimization [18–20], reinforcement learning [21–23], and evolutionary algorithms (EAs) [24–26].

With comprehensive studies on EAs, many efforts have been devoted to solving their weaknesses. For example, an automatic CNN architecture design method with EA [27] was developed to address image classification tasks effectively. A new type of EA-based Haar filter [28] was developed to detect all faces under different conditions, such as illumination variation and rotated faces. A novel evolutionary technique [29] defined the network architecture by a sequence of repeating neurocells, which reduces the searching space complexity. In [30], the authors described the advantages of EAs by controlled comparison of algorithms for architecture search in image classification. Specifically, evolutionary methods have a faster search speed than reinforcement learning approaches, especially in the early stages [30]. Compared with random search strategies, EAs generate smaller architectures and usually complete config searching tasks with competitive performance [30]. Even though EAs play a significant role in the NAS without additional expert knowledge, the fixed-length chromosome genetic encoding strategy may not be suitable for optimizing deep learning hyperparameters, because the performance of CNNs highly depends on the model depth [31]. Compared with the fixed-length chromosome encoding strategy, the variable-length chromosome encoding strategy is qualified for exploring dynamic solution space, which improves locality and significantly reduces redundancy in the representation [32]. For example, VLCGA-NB [33] selects sentiment types in tweets based on the examination of features by a variable-length chromosome EA. A version of the genetic algorithm [34] that uses the variable-length chromosome is proposed to deal with a time series discretization task.

This paper designs an effective and efficient variable-length EA method with a dynamic solution space to diagnose COVID-19 on lung CT images. The contributions of this paper can be summarized as follows:

- A novel EA with variable-length chromosomes is proposed to search hyperparameter settings of CNNs for COVID-19 diagnosis tasks. With the proposed balance operation, including the growth strategy and the shrink strategy, the length of chromosomes may be changed, and the hyperparameter searching space may be altered adaptively, which can significantly reduce dependence on data quality and neural network redundancy without careful pretreatment and expert knowledge.
- A new batch normalization (BN) finding is first presented. The experimental results show that the BN operation indeed boosts CNN performance. However, BN operations should not be used after every convolution operation, and excess BN operations may poorly affect image analysis performance.

## Background and related work

### Deep learning-based diagnosis of COVID-19

Rapid and valid COVID-19 discrimination methods should be designed to control the COVID-19 pandemic and reduce mortality by receiving timely treatment. The traditional nucleic acid test has a nearly 30% false-negative rate [35], which means that negative nucleic acid test results cannot rule out COVID-19 infection. Virus carriers without the correct test result may further spread the disease and produce worse outcomes. In contrast, CT images can effectively diagnose COVID-19. Therefore, the correct recognition of virus-carrying patients through CT images can effectively reduce pressure on hospitals.

To alleviate the burden of medical professionals and efficiently allocate medical resources, many clinicians and scientists aim to develop an accurate COVID-19 diagnosis method based on CT images through novel algorithms. In the field of computer science, machine learning methods have the ability to solve practical difficulties. Moreover, simple machine learning methods have made it possible to accurately diagnose COVID-19 [36, 37]. A sophisticated and effective form of machine learning, known as deep learning, achieves state-of-the-art results in many fields, and CNNs are widely used in deep learning areas to address COVID-19 diagnosis projects. For instance, a tailored CNN called COVID-NET [38] was designed to classify COVID-19 cases from an open and large chest X-ray image data set. A transfer learning-based method with CNNs [39] was adopted to diagnose coronavirus disease in a small X-ray image data set. A new deep anomaly diagnosis model [40] for fast and reliable screening of COVID-19 chest images was developed with a backbone network, a classification head, and an anomaly detection head. The backbone network uses an 18-layer residual CNN pre-trained on the ImageNet data set.

### Batch normalization (BN)

**Fig. 1:**
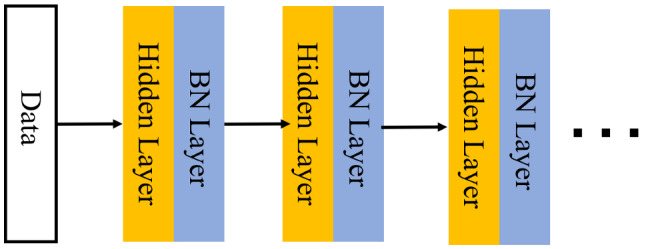
Traditional neural network architecture with the BN operations

Over the last decade, the impressive progress of deep learning hinged on several significant advances in terms of hardware, data sets, and architecture techniques [41–44]. One of the most prominent examples of such advances is BN [45]. In neural networks, the distribution of data has a significant impact on the training phase. In [45], the authors defined internal covariate shift (ICS) as the change in the distribution of network activation due to the change in network parameters during training. To stabilize the layer input distribution, BN operations, which control the first two moments (mean and variance) of these distributions, are introduced after every hidden layer (Fig. 1). Despite their pervasiveness, the effectiveness of the working mechanism is still poorly understood. In addition, there are still controversies about how BN works. In [46], the effectiveness of BN was investigated, and the conclusion was that the connection between BN and ICS performance is tenuous. With more research on BNs, these findings not only help researchers understand more BN details but also help the development and effectiveness of deep learning-related algorithmic toolkits.

### Evolutionary algorithm with a dynamic searching space

Compared with traditional machine learning methods, deep learning methods brilliantly represent data through hidden layers. The deep network structure can easily learn patterns from raw data and abstractly express data with layer-by-layer extraction, which improves task performance. However, this kind of improvement quickly reaches the upper limit. Specifically, the gradient in the backpropagation algorithm will be smaller after more hidden layers. As a result, the performance of deep learning tasks will decrease. For example, the CNN used in AlphaGo has only 13 layers, and the accuracy of the chess move prediction reached 57%. However, when the number of CNN layers was further increased, the prediction accuracy decreased [47].

**Fig. 2:**
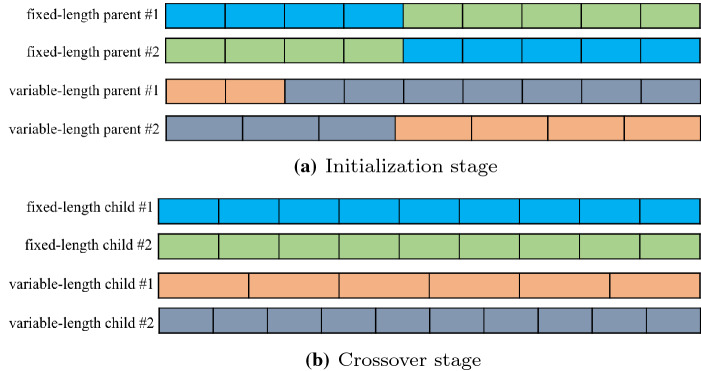
Illustration about fixed-length and variable-length chromosomes

EAs are distinguished global optimizers for black-box functions [48] and can provide a promising set of methods for structural and parametric learning of CNNs [49]. EAs consist of the following essential components: initialization, parent selection, crossover, mutation, and environmental selection. The initialization defines a gene encoding strategy, including genotype [50, 51] and phenotype [29, 52]. In most common EAs, the individuals are represented by a fixed-length encoding [26, 53–55]. Because the depth of a particular CNN greatly affects its performance [9], the variable-length chromosome initialization strategy is suitable for complicated occasions [52]. Figure 2a shows the differences between the fixed-length chromosome strategies and the variable-length chromosome strategies in the initialization stage. Instead of generating the same length parents by the a fixed-length strategy, the length of parents generated by the variable-length strategy is unequal. Tournament selection [53] and fitness proportionate selection [56] are often used for parent selection. The tournament selection method iteratively selects parents with some prespecified threshold values in descending order. In the fitness proportionate selection method, the *i*th individual is chosen with probability $\frac{f(\alpha _i)}{\sum _{j=1}^Nf(\alpha _j)}$, where $\alpha _i\in$ $\{\alpha _i,\ldots ,\alpha _N\}$ and $f(\alpha _i)$ is the fitness of the individual $\alpha _i$. After all the needed parents are selected, the offspring are generated by a crossover operation, which is key to forming new individuals based on exchanging genes. As shown from Fig. 2b, the lengths of the children generated by the fixed-length strategy are equal, but the variable-length strategy obtains the opposite result. The mutation operation generates new chromosomes through mutation probability so that it shows new traits that determine the EA global search ability. Environmental selection addresses elitism by preserving excellent individuals according to fitness evaluation, thus enhancing diversity by initializing new individuals.

## The proposed algorithm

In this section, the proposed evolutionary algorithm with a variable-length chromosome strategy for COVID-19 (EAVL-COVID) diagnosis is described in detail.

### Algorithm overview


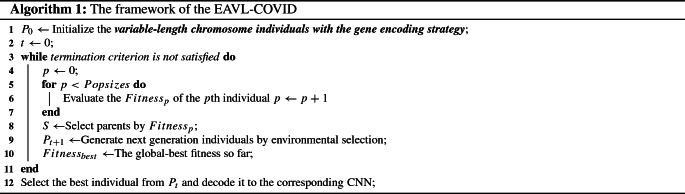


The proposed EAVL-COVID algorithm is mainly composed of two elaborate designs. First, to change the search solution space based on different tasks, EAVL-COVID generates variable-length chromosomes in the initialization step. Second, in EAVL-COVID, the crossover points are chosen randomly, which results in the generated children having chromosomes that are too long or too short after the crossover operation. The neural network, decoded by a long chromosome, may have a deep architecture. In this case, the deep architecture not only leads to overfitting, which reduces the performance but also consumes considerable computational resources. In contrast, the neural network, which is decoded by a short chromosome, cannot solve a difficult task with a plain architecture. As a result, the balance operation with the growth strategy and the shrink strategy is developed to solve unsuitable chromosomes.

Algorithm 1 describes the framework of the proposed EAVL-COVID algorithm, where our contributions and developments based on [52] are highlighted in bold and italics. First, the population is initialized based on the proposed variable-length gene encoding strategy. Specifically, the BN layers are probabilistically added after every convolutional layer (line 1). Second, the evolution begins to take effect until the maximum number of generations is satisfied (lines 3–11). Notably, the random immigrants strategy [57], which is widely used in EA-based NAS methods [26, 53, 58], is adopted to maintain the global diversity level of the population [59]. Finally, the best individual is selected and decoded to the corresponding CNN (line 16) for a specific task.

### Population initialization

**Table 1:** Choice of CNN hyperparameters

Block type	Encode parameters
Convolutional block	The number of input feature maps, the number of output feature maps, the filter size, the stride size, the padding size and the convolutional type
Batch normalization block	Whether to use batch normalization block or not
Pooling block	The kernel size, the stride size and the pooling type
Fully connected block	The number of input neurons and the number of output neurons

A CNN model usually consists of different blocks, such as convolutional layers, BN layers, pooling layers, and fully connected layers. In detail, unlike encoding binary numbers into chromosomes in EAs, the EAVL-COVID algorithm encodes all necessary parameters into four essential blocks presented in Table 1. Particularly, the goal of using the convolutional layers is to extract high-level features with the convolutional filters. With the increase in the number of convolutional layers, the ability to solve challenging tasks can be reinforced. However, the deeper the architecture is, the higher the risks of overfitting and computational consumption. In other words, the proposed variable-length chromosome strategy alerting the solution space can effectively and efficiently solve the particular problem with the appropriate number of convolutional layers.

The steps of initializing a chromosome by EAVL-COVID are described in Algorithm 2. Notably, the number of convolutional layers $n_{\mathrm{c}}$, pooling layers $n_{\mathrm{p}}$ and fully connected layers $n_{\mathrm{f}}$ are chosen from the predefined ranges (line 2). Specifically, the convolutional layers and the pooling layers are chosen by the once coin-tossing probability (lines 3–22). If the convolutional layers are chosen, with or without a BN layer should be randomly determined (lines 11–19). After repeatedly choosing $n_{\mathrm{c}}$ convolutional layers and $n_{\mathrm{p}}$ pooling layers, $n_{\mathrm{f}}$ fully connected layers are added to the end of the chromosome by encoding the necessary information, such as the number of neurons, which are predefined in advance (lines 23–27).

After all those steps are finished, the EAVL-COVID algorithm obtains one integrated individual. With the same approach, a population with variable-length chromosome individuals is generated.
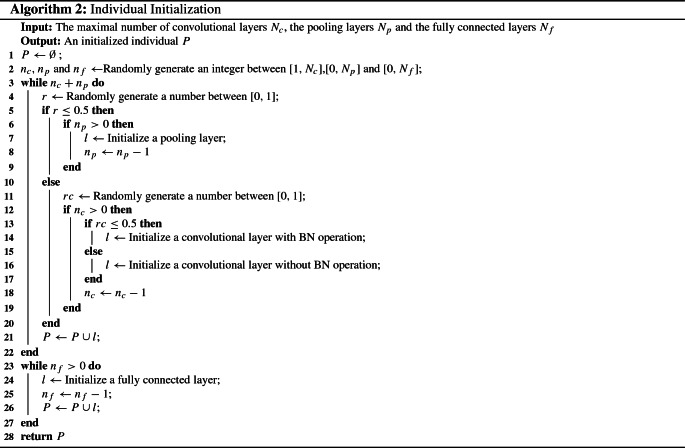


### Parents selection and chromosome crossover

**Fig. 3:**
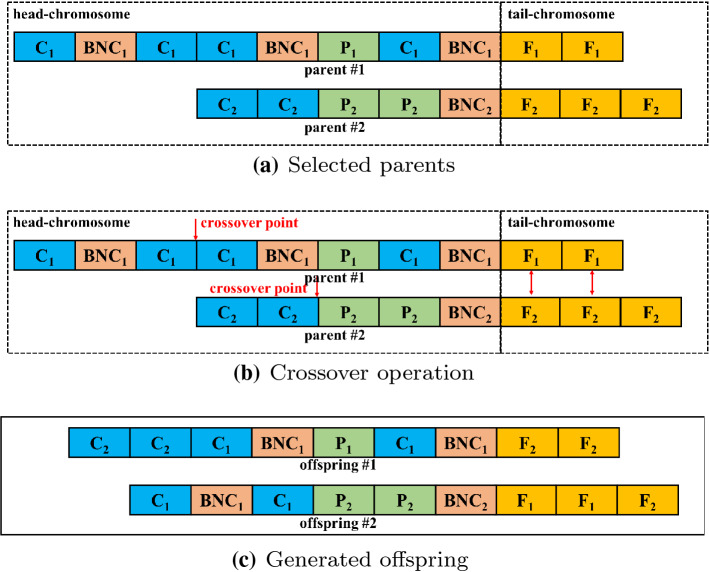
Example to illustrate the crossover operation with variable-length chromosomes. In this example, parents are split into two parts (see **a**). The head-chromosome is made up of C, BNC and P. All F are all in the tail-chromosome and the first F in each parent are aligned at the same position. **b** Fully shows the crossover operation. For head-chromosome, a crossover point is random selected in each parents separately. After the head-chromosomes before crossover points are exchanged, the F in the same position are exchanged, respectively. **c** Describes the offspring generated by the selected parents through crossover operation

The steps for selecting parents and generating offspring are given as follows:

1. select parents through a fitness-based process, where fitter solutions are typically from the population $P_t$;
2. divide the chromosome of each parent into two parts, because the crossover operation is different;
3. use a crossover strategy on the selected parents to generate offspring.

First, EAVL-COVID uses a fitness-based method to select two parents, which means that the more suitable the parent is for the COVID-19 classification task, the higher the probability of being selected.

Second, each selected parent chromosome is divided into a head chromosome and a tail chromosome, because the crossover operation for each part is different. The head chromosome includes convolutional blocks (C), batch normalized convolutional blocks (BNC), and pooling blocks (P). The tail chromosome only involves fully connected blocks (F).

Third, the crossover strategy is implemented on the selected parents to generate offspring. Figure 3 shows the crossover operation intuitively. In particular, the crossover points are first randomly generated in head chromosomes. Then, the head chromosomes before the generated points are exchanged. After performing the crossover strategy on the head chromosome, all the blocks in the tail chromosome at the same positions perform a crossover strategy. Due to individuals possessing variable-length tail chromosomes, if the F in one parent mismatches those at the same positions as the other parents, those F will not participate in crossover processing. After the above steps are finished, offspring that have information on both parents are generated.

### Balance operation

**Fig. 4:**
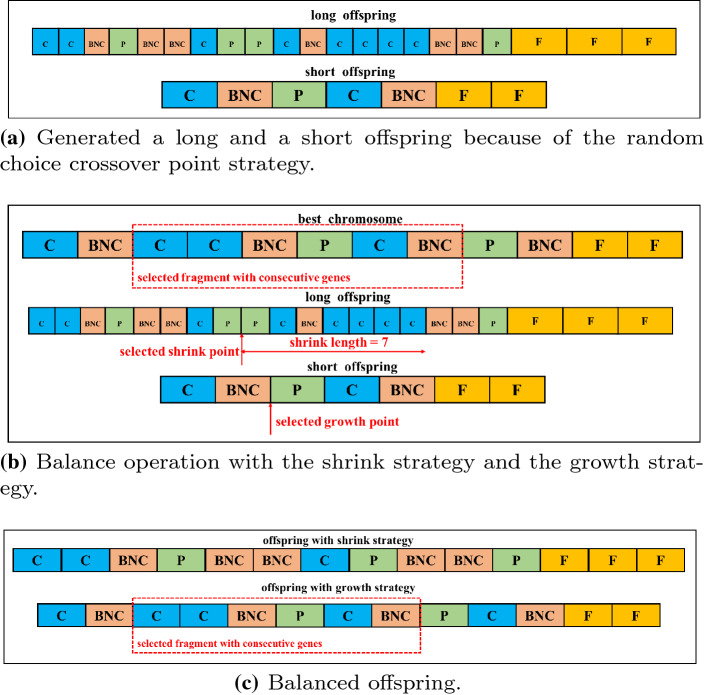
Example shows the balance operation to deal with a long offspring and a short offspring generated by the proposed crossover operation. The numbers of units in head-chromosomes $len\_offspring\_head$ are 18 and 5, respectively (**a**). **b** Detailedly give the steps of the balance operation, where $len\_best\_head = 10$ and $\varphi =6$. Due to the long offspring $len\_offspring\_head-len\_best\_head>\varphi$, the shrink strategy choices a shrink position $index\_shrink=8$ and the shrunken length $len\_shrink = 7$; For the short offspring, where $len\_offspring\_head<\varphi$, a fragment with $\varphi$ consecutive genes is selected in global-best chromosome, followed by a growth point $index\_growth=2$. Finally, the selected fragment is inserted after the growth point. **c** Gives the picture that the balanced offspring with both $len\_offspring\_head=11$ after the balance operation

As the crossover points are chosen randomly, the lengths of all offspring chromosomes are diverse. If the chromosome is too long, a deep neural network encoded by this chromosome may be generated. Even if the complicated architecture may help accomplish a difficult task, it may also cause overfitting or consume too many computational resources. In contrast, if the chromosome is too short, the plain neural network may be unequipped with the ability to solve a difficult problem. To address those shortcomings caused by the variable-length chromosomes, the balance operation with the shrink strategy and the growth strategy, which replaces the mutation operation in original EAs, is proposed in our work. Figure 4 expressly describes the balance operation as an example.

More specifically, a shrink strategy is taken if $l\ge \varphi$, where *l* is the difference between the length of the offspring’s head-chromosome $len\_offspring\_head$ and the individual’s head-chromosome with global-best fitness $len\_best\_head$.

$\varphi$ is a hyperparameter for controlling the maximum depth of networks. First, the shrunken length $len\_shrink$ of the shrink strategy is determined by a probabilistic method. Given a normal distribution ${\mathcal {N}}(l,1)$, each element $i\in [1,\ldots ,l]$ is given a value called $\rho _i$, where

1 $$\begin{aligned} \rho _i \sim {\mathcal {N}}(i;l,1). \end{aligned}$$

After normalizing all $\rho _i$, the choice probability of $len\_shrink$ is obtained and the choice probabilities determine $len\_shrink$. Then, a shrink point $index\_shrink$ is generated in the position range $[1, len\_offspring\_head-len\_shrink]$. A balanced offspring is generated with $index\_shrink$ and $len\_shrink$.

However, if $len\_offspring\_head<\varphi$, the short offspring will be performed with a growth strategy. Specifically, a growth point $index\_growth$ is generated within the position range $[1,len\_offspring\_head]$. Because of the global-best fitness individual with the excellent genes solves a particular job well, a fragment with $\varphi$ consecutive genes is selected from the global-best fitness individual. Then, the selected fragment is inserted into the short offspring at the generated position $index\_growth$.

In [52], even though variable-length chromosomes are generated in the population initialization phase, the chromosome length is fixed during evolution. However, the proposed balance operation with the shrink strategy and the growth strategy vary the length of chromosomes during evolution, which may exceed the predefined range. This means that the search space is dynamic, and an optimal CNN architecture could be principally found to address complex problems based on different data sets. 
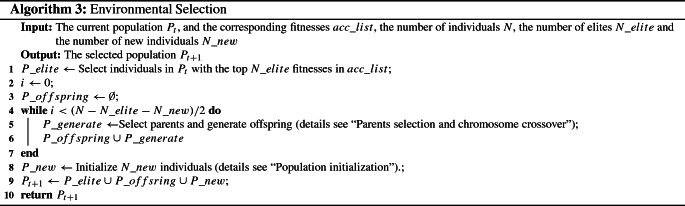


### Environmental selection

To ensure the elitism and diversity of the evolutionary population, the next generation consists of the elites in the current population, the offspring generated by the crossover operation and the balance operation, and new individuals generated by initialization. The environmental selection is shown in Algorithm 3. The proposed EAVL-COVID algorithm chooses the top $N\_elite$ elites with the fitnesses in descending order from the current population to ensure the elitism. Then, $N\_new$ individuals are initialized to help the EA avoid local optimization and increase the opportunities for better results.

## Experimental design

In this section, to examine the performance of the proposed algorithm on the COVID-19 classification task, the data set, peer competitors and parameter settings are provided, which are documented in “COVID-CT dataset”, “Peer competitors” and “Parameter settings”, respectively.

**Table 2:** Detailed information about COVID-CT

	COVID-19	Non-COVID-19
Train set	88	231
Test set	173	168
Val set	88	64
Total	349	463

### COVID-CT data set

The COVID-CT data set [60] has 349 CT images labelled as being positive for COVID-19 from 216 patients, which were collected from COVID-19-related papers and 463 negative CT images from four biomedical CT scan data sets. This data set was confirmed by a radiologist who performed the diagnosis and treatment of a large number of COVID-19 patients during the virus outbreak in Wuhan. Detailed information about the data set is shown in Table 2.

**Fig. 5:**
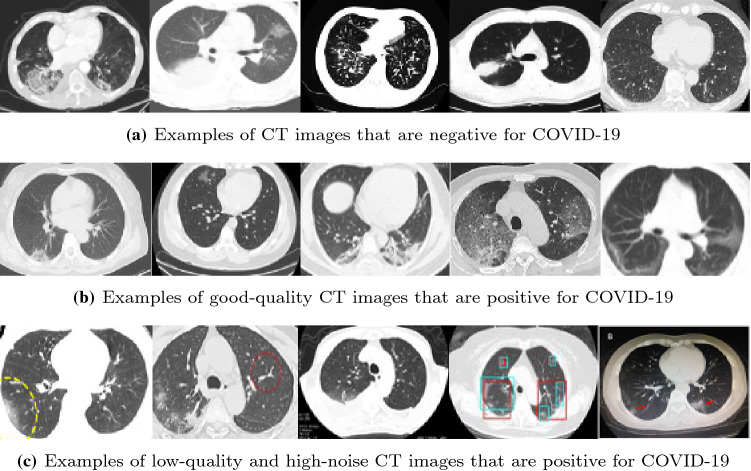
Examples of CT images from COVID-CT data set

Because the original clinical findings of COVID-19 images were obtained from papers, the sizes of these images are diverse. In addition, many positive COVID-19 CT images are degraded, so the quality of those images is unpromising. Figure 5 illustrates examples of CT images from the COVID-CT data set. There are numerous invalid marks on the labelled positive COVID-19 lung images, which may render the diagnosis decisions less accurate. Moreover, the size of the COVID-CT data set, compared with other computer vision tasks in the deep learning area, is severely insufficient. As a result, the CT images must be preprocessed, but this process is extremely time consuming, and the architecture used for training must be elaborately designed.

### Peer competitors

To demonstrate the effectiveness of the proposed EAVL-COVID algorithm, we first experiment on randomly initialized networks with different backbones as baselines, and pre-trained them on ImageNet. The networks include VGG-16 [9], ResNet-18 [10], ResNet-56 [10], DenseNet-121 [61], DenseNet-169 [61], EfficientNet-b0 [62], and EfficientNet-b1 [62]. The BN operation is used through all models and the binary cross-entropy serves as the loss function.

As a basic network, VGG-16 has good classification performance and adaptability to many data sets and provides meaningful performance for the COVID-19 classification task on the COVID-CT data set. As the depth of the network increases, the model obtains better representation ability. However, with the deeper architecture, the training accuracy tends to be flat, and the training error becomes larger. ResNets use the skip connection to solve the optimization problems when the number of hidden layers is deepened, such as ResNet-18 and ResNet-56. DenseNet realizes feature reuse through the connection of features on the channel. These features allow DenseNet-121 or DenseNet-169 to achieve better performance than ResNets with fewer parameters and computational costs. To pursue better accuracy and efficiency, EfficientNet balances all dimensions of network width, depth, and resolution during convolutional neural network scaling by neural architecture search. Unlike the EA used by the proposed EAVL-COVID algorithm, EifficientNet introduces a factorized hierarchical search space and solves the Pareto optimal solution of the multi-objective search problem by reinforcement learning [63].

### Parameter settings

All the parameters are set based on the conventions in the EA communities and deep learning. The proposed EAVL-COVID algorithm is implemented in PyTorch, and each copy of the code runs on a computer equipped with two identical GTX2080Ti GPU cards.

First, in the EA initialization, the population size is set to be 50. The range of the convolutional layer depth with a $3 \times 3$ kernel is set to be [5, 8], and those convolutional layers have a 50% chance of performing a BN operation. The number of neurons in each convolutional layer is set to be [50, 100]. The maximal depth of the pooling layers whose kernel size and stride size are both set to be 2, and fully connected layers with [100, 500] neurons are set to be 5 and 4, which is believed to be sufficient to solve the benchmark data sets employed at the beginning of the classification task. In the crossover step, the probability of the crossover operation is set to be 0.9. In the balance step, the threshold value $\varphi$, which determines the choice of the growth strategy or the shrink strategy, is set to be 4. In the environmental selection step, 10 elites are preserved to the next generation, and 10 new individuals are generated by a gene encoding strategy. To weight the performance of each individual, the chromosomes are decoded into neural networks that are trained with the following process. In the training phase, the Adam optimizer is used with an initial learning rate of 1e−4 and a batch size of 16. Due to the time consumption of the proposed method, the training epoch of each individual is set to be 100, which is inadequate but workable during the EA procedure. Due to the stochastic nature of the proposed EAVL-COVID method, the chromosome with the best classification accuracy is generated after 20 generations. To achieve the truthful performance, the best chromosome is decoded and trained by another 1000 epochs.

## Experiment

**Fig. 6:**
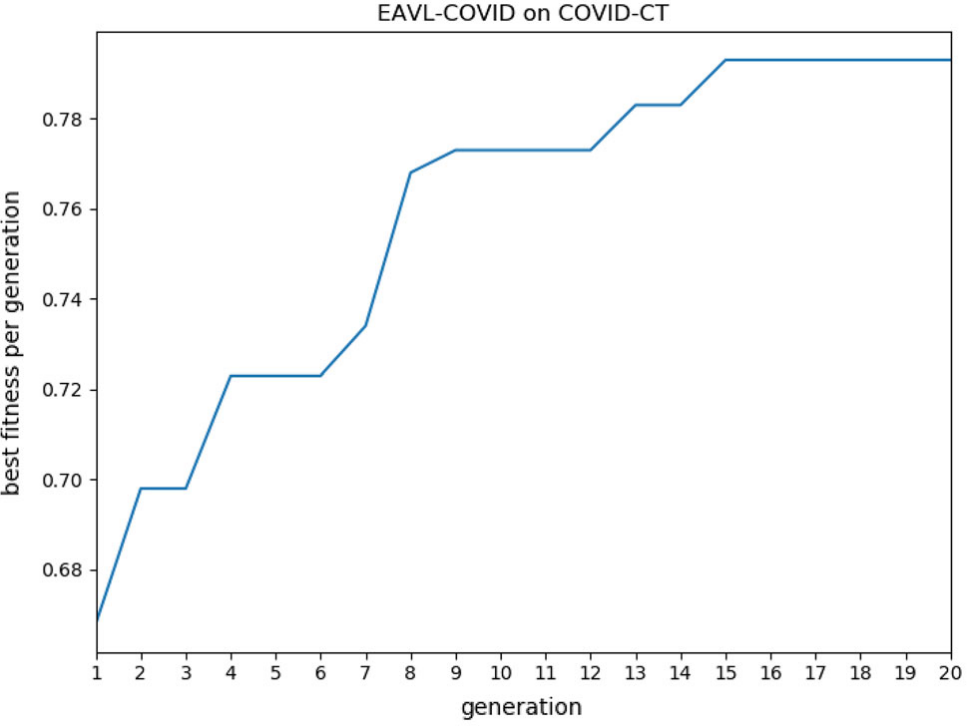
Evolution curve of EAVL-COVID on COVID-CT

To perform the COVID-19 classification task on the COVID-CT data set, the proposed EAVL-COVID algorithm follows three steps. First, the generation number is set to 20. The evolution curve of the proposed EAVL-COVID algorithm is shown in Fig. 6. Second, after 15 generations, the chromosome with the global-best fitness is generated. Third, this chromosome is decoded to the convolutional neural network, which is used for the COVID-19 classification task. To investigate the character of the BN operation, the best chromosome is decoded into three architectures: ORIGIN, VARIANT#1 and the VARIANT#2. ORIGIN represents the architecture searched by the EAVL-COVID algorithm without any modification. The three architectures on the COVID-CT data set are illustrated in Table 3. As shown in Table 3, BN operations are not used in the three convolutional layers of the ORIGIN. They are conv-79 located at the 4th layer, conv-99 located at the 7th layer, and conv-58 located at the 9th layer. VARIANT#1 adds a BN operation after those three convolutional layers, where the ORIGIN is missing. In addition, VARIANT#2 removes all BN operations of the ORIGIN.

To evaluate the performance of the proposed EAVL-COVID algorithm, seven networks for the COVID-CT data set are chosen as the baseline. We first experiment on the randomly initialized networks and then the pre-trained networks on a large-scale data set. The quantitative results aredocumented in Table 4 and the best performance is highlighted in bold.

**Table 3:** ORIGIN searched by EAVL-COVID and its variants on the COVID-CT data set

The ORIGIN searched by EAVL-COVID and its variants		
ORIGIN	VARIANT#1	VARIANT#2
Inputs		
maxpooling	maxpooling	maxpooling
conv-91	*conv-91	conv-91
conv-69	*conv-69	conv-69
conv-51	*conv-51	conv-51
conv-79	*conv-79	conv-79
conv-72	*conv-72	conv-72
conv-53	*conv-53	conv-53
conv-99	*conv-99	conv-99
maxpooling	maxpooling	maxpooling
conv-77	*conv-77	conv-77
conv-58	*conv-58	conv-58
maxpooling	maxpooling	maxpooling
FC-347		
FC-344		
FC-256		
FC-193		
softmax		

*The convolutional layer followed a BN layer

### Evaluations on randomly initialized networks

To fairly evaluate the performance of EAVL-COVID and backbones on the COVID-CT data set, the Kaiming initialization strategy [64] is adopted to initialize the weights randomly.

**Table 4:** Performance comparison between randomly initialized networks (RAND.) and ImageNet pretrained networks (PRET.)

		Parameter numbers	RAND.	PRET.
BASELINE [65]	VGG-16	138,357,544	0.632	0.773
	ResNet-18	11,689,512	0.652	0.734
	ResNet-50	25,557,032	0.687	0.775
	DenseNet-121	7,978,856	0.773	0.814
	DenseNet-169	14,149,480	0.807	**0.842**
	EfficientNet-b0	5,288,548	0.695	0.774
	EfficientNet-b1	7,794,184	0.726	0.797
EAVL-COVID	VARIANT#2	808,222	0.680	0.663
	VARIANT#1	810,565	0.749	0.738
	ORIGIN	810,093	**0.813**	0.815

As seen in the performance (columns marked with “RAND.”) for different neural networks trained with random initialization in Table 4, the ORIGIN evolved by EAVL-COVID achieved 81.3% accuracy and obtained the best performance among all competitors. Amazingly, the evolution results of ORIGIN outperform the performance of DenseNet-169 with skip connection operation. The following two reasons can explain the result. First, the original clinical findings of COVID-19 images were obtained from published papers. Many positive COVID-19 CT images are degraded with additional marks. This drawback of COVID-CT reduces the classification accuracy without preprocessing. The proposed variable-length chromosome strategy improves locality and significantly reduces redundancy in the representation. In conclusion, it is difficult for statistical scientists to design a suitable architecture without discreetly processing data. The second reason is that the COVID-CT is a data set with only 349 positive COVID-19 CT images. The architecture of the neural network deeply affects the accuracy of the classification task. Even though DenseNet introduces skip connections to avoid overfitting, the sophisticated architecture still fails in the classification task on the COVID-CT data set. Without unique domain knowledge designs, there are many obstacles to perfectly finishing a particular goal by transferring models that have excellent performance on other tasks in a short time. However, the proposed EAVL-COVID algorithm, which works automatically, with a dynamic search space and without any preprocessing or manual parameter settings, is perfectly suitable for this complicated task.

In addition, the BN layers improve the performance of image classification tasks. Compared with VARIANT#2 based on the COVID-CT data set, ORIGIN and VARIANT#1, which have BN operations, improve accuracy by 13.3 and 6.9%, respectively. Surprisingly, the experimental results show that the classification accuracy deteriorates by 6.4% if all convolutional layers are followed by BN layers.

### Evaluations on pre-trained networks

In deep learning, many training tricks boost classification performance. The pre-train strategy is largely used for general knowledge discovery with no training phase or use of labels to generate high-level representations, which helps much research to achieve state-of-the-art results over various classification benchmarks. After evaluating the performance of different neural networks on random initialization, we further investigate the performance of networks pre-trained on ImageNet. Columns marks with “PRET.” in Table 4 show the classification accuracy on COVID-CT after training the networks on the ImageNet data set.

In certain cases, the pre-training strategy improves the task performance of the networks. For example, a 14.3% improvement in accuracy appears in VGG-16, which has the worst performance in the random initialization. After transfer learning, the performance of VGG-16 exceeds the performance of ResNet-18 and ResNet-50. The reason is apparent. Compared with sophisticated architectures designed for preventing overfitting, VGG-16 speeds up convergence and improves the ability to extract features through pre-trained weights. In contrast, the effect of the pre-training strategy on the ORIGIN is marginal. The reason is that the proposed EAVL-COVID algorithm has found a good solution for a specific task. The variable-length chromosome strategy reduces the architecture redundancy and avoids overfitting in large networks. Without any preprocessing operation, the ORIGIN’s performance can compete with DenseNet-169, whose accuracy was the highest at 84.2% after the pre-training strategy.

Similar results appear in the pre-trained network. Among VARIANT#1 and VARIANT#2, ORIGIN achieves the best performance on the COVID-CT data set. In conclusion, the BN operation may reduce the performance in some situations, so it is unnecessary to perform BN operation after every convolution operation. This interesting viewpoint was found by experimental results, and to the best of our knowledge, is summarized for the first time.

### Ablation experiments

The idea of the variable-length strategy is hyper-heuristic optimization methods that construct a heuristic from several low-level heuristics to solve common problems in operations research. In searching for the best heuristic, the optimal number of low-level heuristics is unknown a priori, so a variable-length chromosome is designed to explore heuristics made from low-level heuristics. Similarly, a variable-length chromosome can be used to search the number of hidden layers, since the number of hidden layers in different data sets for optimal coverage performance is unknown a priori. With the proposed growth strategy and shrink strategy, the proposed variable-length chromosome representation adaptively alters the searching space and may find the optimal number of hidden layers based on different data sets.

To fully understand the effects of the variable-length strategy and investigate the usage rules of BN operation, three competitors are chosen to conduct ablation studies.

- **VGG16BN:** A classic CNN human-made architecture with BN operation.
- **Random Search(RS):** An NAS method that randomly searches for the CNN architecture in a similar searching space as the initial definition of the proposed EAVL-COVID algorithm, and the searching space is static during the whole process. Compared with EAVL-COVID, the RS does not determine whether to use BN. Basically, each convolutional layer is followed by the BN operation. To be fair, the RS generates 1000 architectures on each data set used.
- **EVO-CNN:** An EA-based NAS method evolutionarily searches for the CNN architecture in a searching space similar to the initial definition of the proposed EAVL-COVID algorithm, and the search space is static during the whole process. In addition, the BN operation is adopted by each convolutional layer.
- **EAVL-COVID:** The proposed EA-based NAS method searches for the CNN architecture in a dynamic searching space. The initially defined searching space is demonstrated in “Parameter settings”.

**Table 5:** Performance of ablative experiments on different data sets

	COVID-CT		CIFAR10		CIFAR100		STL10	
	Accuracy	# of layers	Accuracy	# of layers	Accuracy	# of layers	Accuracy	# of layers
VGG16BN	0.632	16	0.611	16	0.602	16	0.589	16
RS	0.618	11	0.583	12	0.512	12	0.544	12
EVO-CNN	0.758	10	0.784	12	0.587	12	0.582	12
EAVL-COVID	**0.813**	13	**0.899**	11	**0.648**	15	**0.590**	13

**Table 6:** Performance of EAVL-CNN and its variants on different data sets

Optimizer	Architecture	CIFAR10	CIFAR100	STL10
SGD	VARIANT #2	$0.615\pm 0.0027$	$0.525\pm 0.0468$	$0.534\pm 0.0127$
	VARIANT #1	$0.678\pm 0.0018$	**0.628** ± **0.0219**	$0.560\pm 0.0153$
	ORIGIN	**0.885** ± **0.0014**	$0.624\pm 0.0441$	**0.576** ± **0.0237**
Adadelta	VARIANT #2	$0.536\pm 0.0042$	$0.545\pm 0.331$	$0.537\pm 0.0094$
	VARIANT #1	$0.628\pm 0.0038$	$0.619\pm 0.183$	$0.559\pm 0.0082$
	ORIGIN	**0.862** ± **0.0046**	**0.640** ± **0.257**	**0.584** ± **0.0132**
RMSprop	VARIANT #2	$0.572\pm 0.0027$	$0.553 \pm 0.0354$	$0.526\pm 0.0157$
	VARIANT #1	$0.618\pm 0.0020$	$0.608\pm 0.0192$	$0.563\pm 0.0091$
	ORIGIN	**0.863** ± **0.0018**	**0.655** ± **0.0223**	**0.581** ± **0.0095**
Adam	VARIANT #2	$0.566\pm 0.0031$	$0.541\pm 0.0143$	$0.538\pm 0.0111$
	VARIANT #1	$0.625\pm 0.0019$	$0.607\pm 0.0224$	$0.561\pm 0.0092$
	ORIGIN	**0.881** ± **0.0025**	**0.642** ± **0.0117**	**0.588** ± **0.0075**

To investigate the advancements of the proposed EAVL-COVID algorithm with the variable-length chromosome strategy during the crossover and mutation stages, ablation experiments are performed on four data sets.

- **COVID-CT:** A small data set with low-quality and high-noise images, and there are two classes in the COVID-CT.
- **CIFAR10:** A classic data set in the computer version community, and each class contains 10 times more images than the COVID-CT.
- **STL10:** A data set with high-resolution images, which means that the high dimensionality of data increases the computational complexity of algorithms owing to the effect of noise [66, 67]. Compared with CIFAR10, each category contains fewer labelled training examples.
- **CIFAR100:** A data set with 100 classes that greatly exceed the number of classes in COVID-CT, CIFAR10, and STL10.

To fairly evaluate the performance of ablation experiments, only the Kaiming initialization strategy is adopted to initialize the weights randomly. Moreover, no preprocessing or post-processing techniques are adopted on the human-made architecture, the autosearched architectures and data sets.

The results of ablative experiments on different data sets are describled in Table 5 and the best performance is highlighted in bold. It is clearly shown in Table 5 that the proposed EAVL-COVID algorithm achieves excellent performance on all chosen data sets. In detail, EAVL-COVID obtains significant advantages on the COVID-CT and CIFAR10 data sets. On COVID-CT, the accuracy of EAVL-COVID is 18.1% higher than that of the classical human-made architecture CNN. Moreover, the proposed EAVL-CNN also ranks first among the NAS methods. Compared with the performance of EAVL-CNN on COVID-CT, the accuracy rates of RS and EVO-CNN are less than 19.5 and 5.5%, respectively. A similar phenomenon can also be observed from the CIFAR10, CIFAR100, and STL10 data sets. Notably, the RS fails to achieve good performance on all data sets due to the ineffective search strategy and the limited number of generated architectures.

As shown in Table 5, EAVL-COVID generates different numbers of layers on different data sets, and the generated depth of the architectures may exceed the predefined value, i.e., the searching space is dynamic. Specifically, the number of hidden layers is set to [5, 8], and the number of fully connected layers is [1, 4]. As a result, the maximal depth of the architectures searched by the NAS methods with static searching space is 12. However, the proposed EAVL-COVID algorithm can obtain CNNs exceeding predetermined depths. In the experiments, the depths of the architectures searched on COVID-CT, CIFAR100, and STL10 were 13, 15, and 13, respectively. Based on the results shown in Table 5, the conclusion can be made that the optimal priori about the depth of the architectures on different data sets can be automatically obtained with the variable-length strategy with the proposed crossover strategy and mutation strategy.

To investigate whether it is necessary to use the BN operation after each convolutional layer, more experiments are performed based on the architectures searched by the proposed EAVL-COVID algorithm on CIFAR10, CIFAR100, and STL10. First, the architecture searched by the EAVL-COVID is expanded into ORIGIN, VARIANT #1, and VARIANT #2. Second, three additional optimizers are selected to verify if the observation still holds true. Each architecture is conducted five times with the Kaiming initialization strategy and trained for 1000 epochs. The experimental results are documented in Table 6. The best performance of the architectures is highligthed in bold according to the optimizers and data sets. A similar conclusion about BN can also can be found in Table 6. Compared with VARIANT #2 and VARIANT #1 based on the CIFAR10, the best overall test accuracies are all found by the ORIGIN. In detail, the ORIGIN achieves a test accuracy of 0.885 ± 0.0014 with SGD, 0.862 ± 0.0046 with Adadelta, 0.863 ± 0.0018 with RMSprop, and 0.881±0.0025 with Adam. Moreover, the results on the CIFAR100 and the STL10 have the similar circumstances. Notably, a special situation appears in the CIFAR100 with SGD. Even though the VARIANT #1 achieves the best result, the performance of the ORIGIN is still competitive with a high standard deviation. In conclusion, it is not necessary to perform BN operations after every convolutional layer. To the best of our knowledge, this is the first time this interesting viewpoint has been experimentally identified.

To investigate whether it is necessary to use the BN operation after each convolutional layer, more experiments are performed based on the architectures searched by the proposed EAVL-COVID algorithm on CIFAR10, CIFAR100, and STL10. First, the architecture searched by the EAVL-COVID is expanded into ORIGIN, VARIANT #1, and VARIANT #2. Second, three additional optimizers are selected to verify if the observation still holds true. Each architecture is conducted five times with the Kaiming initialization strategy and trained for 1000 epochs. The experimental results are documented in Table 6. The best performance of the architectures is highligthed in bold according to the optimizers and data sets. A similar conclusion about BN can also can be found in Table 6. Compared with VARIANT #2 and VARIANT #1 based on CIFAR10, the best overall test accuracies are all found by ORIGIN. In detail, ORIGIN achieves a test accuracy of 0.885 ± 0.0014 with SGD, 0.862 ± 0.0046 with Adadelta, 0.863 ± 0.0018 with RMSprop, and 0.881 ± 0.0025 with Adam. Moreover, the results on CIFAR100 and STL10 have similar circumstances. Notably, a special situation appears in CIFAR100 with SGD. Even though VARIANT #1 achieves the best result, the performance of ORIGIN is still competitive with a high standard deviation. In conclusion, it is not necessary to perform BN operations after every convolutional layer. To the best of our knowledge, this is the first time this interesting viewpoint has been experimentally identified.

## Conclusion and future work

The objective of the paper was to develop a new evolutionary approach to automatically evolve the CNN architecture for the COVID-19 classification task. This goal was successfully achieved by the proposed variable-length chromosome strategy with the growth operation and the shrink operation, which are suitable for classification tasks with a dynamic searching space. With the detailed investigation on random initialization networks and pre-trained networks, the experimental results showed that the proposed EAVL-COVID algorithm significantly outperforms most competitors on the COVID-CT data set in terms of their best classification performance. The effectiveness of the EAVL-COVID algorithm is fully verified. Without any extra designs, the performance of the network searched by EAVL-COVID is still competitive, which can save time and human resources. Furthermore, an important conclusion about BN operation is drawn in the related research area. The experimental results showed that it is not necessary to perform a BN operation after every convolution layer, because it may reduce the classification accuracy. In the method-applied stage, the proposed algorithm is successfully applied to the COVID-19 diagnosis task. The model optimized by EAVL-COVID reached the highest classification performance, 1.8% higher than the score obtained by DenseNet-169 with sophisticated skip connections and dense connections. In the future, we will invest efforts in combining the variable-length chromosome strategy and skip connection, which will widen the application area of EAVL-COVID. In addition, research on the working mechanism of the BN operation will be further included. Moreover, we will investigate the phenomenon that pre-training on ImageNet does not significantly improve the performance of the architecture searched by the evolutionary algorithm.

## Declarations

### Availability of data and material

All data generated or analyzed during this study are included in this published article.
